# Patient Acceptability and Technical Reliability of Wearable Devices Used for Monitoring People With Parkinson Disease: Survey Study

**DOI:** 10.2196/63704

**Published:** 2025-03-25

**Authors:** Tasmin Alanna Rookes, Amit Batla, Megan Armstrong, Gareth Ambler, Kate Walters, Anette Schrag

**Affiliations:** 1 Research Department of Primary Care and Population Health, University College London, London, United Kingdom; 2 Department of Clinical and Movement Neurosciences, UCL Queen Square Institute of Neurology, University College London, London, United Kingdom; 3 Department of Statistical Science, University College London, London, United Kingdom

**Keywords:** Parkinson disease, feasibility, remote monitoring, Parkinson, acceptability, reliability, wearable devices, wearable, self-management, quantitative assessments, quantitative, qualitative, monitoring, patient

## Abstract

**Background:**

Parkinson disease is a progressive neurodegenerative disorder with complex motor and nonmotor symptoms. To assess these, clinical assessments are completed, providing a snapshot of a person’s experience. Monitoring Parkinson disease using wearable devices can provide continuous and objective data and capture information on movement patterns in daily life.

**Objective:**

The aim of the study is to assess patient acceptability and technical reliability of 2 wearable devices used in clinical trials (ActivInsights and Axivity AX3).

**Methods:**

Participants in a feasibility study testing a self-management toolkit (PD-Care) optionally wore a wearable device for 1 week, providing feedback through an open- and closed-question survey conducted over the telephone about the acceptability of wearing the device. The closed questions used a Likert scale from 1 to 5 (with 1=strongly agree and 5=strongly disagree) asking whether (1) the device was comfortable to wear, (2) the device was easy to put on, (3) the device was easy to wear, (4) the device was embarrassing to wear, and (5) if they were happy to wear the device for longer than 7 days. Differences in acceptability between devices were analyzed using Mann-Whitney *U* tests and Wilcoxon matched pairs signed rank tests. These were followed by open-ended questions asking (1) How did you find wearing the device? (2) How did you find putting the device on? (3) Did you take it off and why? (4) What was your overall impression? (5) Did you prefer the wrist- or trunk-worn device and why (Axivity AX3 only)?

**Results:**

A total of 22 of 32 (69%) participants offered the device agreed to wear it. There were no significant differences in the demographic characteristics between those monitored and those who chose not to be. Acceptance with both devices was generally good. The ActivInsights device was more acceptable than the wrist- and trunk-worn Axivity AX3 devices, as more participants found it to be comfortable (n=15, 100% vs n=5, 71%; *P*=.02 and n=4, 57%; *P*=.004, respectively), easy to wear (n=15, 100% vs n=6, 86%; *P*=.048 and n=3, 43%; *P*=.004, respectively) and would wear for more than 7 days (n=13, 87% vs n=4, 57%; *P*=.02 and n=1, 14%; *P*<.001, respectively). The trunk-worn Axivity AX3 device had the lowest acceptance rates, but there were no statistical differences in acceptability between the wrist- and trunk-worn Axivity AX3 devices (all *P*>.05). There were issues with battery life and recording errors in 3 of 14 (21%) Axivity AX3 devices and upload failures in 3 of 15 (20%) ActivInsights devices.

**Conclusions:**

Acceptability of wearables for monitoring Parkinson was satisfactory, especially when wrist-worn, although a few participants experienced difficulties in correct use, and there were some errors with the data upload.

## Introduction

Parkinson disease (PD) is a neurodegenerative chronic disease, affecting around 145,000 people in the United Kingdom in 2018, equivalent to approximately 1 adult in every 350 [1]. The currently accepted monitoring of PD symptoms in clinical practice and trials is based on interviews and validated clinical scales and questionnaires [2]. However, the use of patient- and clinician-rated scales is resource-intensive, and the results limited by subjectivity and inaccuracy and are either one-off assessments or dependent on the patient’s recollection [3]. Continuous and accurate assessment of PD features, in both clinical practice and trials, would provide more relevant information and overcome the limitations of clinical scales and questionnaires through real-world data. With the increasing burden of care and limited medical resources, new assessment and monitoring approaches have the potential to improve clinical care and patient outcomes [4]. There are now a range of body-worn sensors using accelerometers or gyroscope devices (henceforth called wearable devices) that have been developed and validated to monitor parkinsonian clinical features, such as motor fluctuations, dyskinesia, tremor, bradykinesia, freezing of gait, or gait disturbances [2]. However, relatively little evidence exists on their use, acceptability, and feasibility in clinical practice and trials with patients with PD. In addition, the physical, technical, or practical limitations on prolonged use for real-time recording, uploading, and analysis of movement data are not clear [5].

The National Institute of Health and Care Excellence has conditionally recommended some wearable devices for remote monitoring of PD. However, the exact role, indication, and type of device is unclear, and evidence on the use of wearable devices is limited [6]. The cost, burden on patients and services, and effectiveness to improve clinical outcomes have not yet been assessed. National Institute of Health and Care Excellence has therefore recommended the collection of real-world evidence on devices that monitor people with PD [7] to enable the clinical and cost-effectiveness of the technologies to be fully assessed, ahead of further implementation into clinical practice. In practice, wearable devices are relatively rarely used due to several factors, such as unfamiliarity, unclear indications, cost, acceptability, and technical issues. Furthermore, although digital measures are considered to have high potential for future use to more accurately detect meaningful change in clinical trials, little data exist on their use and acceptability in clinical trials.

Data collection regarding utility and acceptability has largely been informal and focused on wearable devices in general [5]. A study in Finland reported that 88.9% (32/36) of patients with PD using a wearable device thought it was very easy or easy [8]. In the United States, the recent WATCH-PD study [9] explored the acceptability of wearables in people with PD and control participants over a 2-year window and found that participants with PD had generally positive views of the wearables, which were comparable to healthy controls. Conducting qualitative interviews with people with PD who had used multimodal sensors, including a wearable device, the wrist-worn wearable was the least acceptable, in comparison to cameras and passive ambient sensors [10]. Another small study explored perceptions of wearing devices in the home versus in public and found no differences in acceptability between these settings and acceptance of long-term symptom monitoring through wrist-worn devices [11]. However, these specific studies are likely to have recruited participants willing to wear a device, so there is a lack of real-world evidence from patients with PD wearing these devices in home and social settings. Eliciting opinions from patients on wearing such devices in real-life settings may provide important information to facilitate the implementation of wearables into clinical practice.

Here, we have used the opportunity to explore the views of participants taking part in a feasibility study from a large randomized controlled trial of a self-management tool for people with PD in the National Health Service (NHS) to obtain such information in a real-life setting. The objective of this study was to explore the acceptability and technical reliability of 2 wearable sensors that are commonly used by people with PD.

## Methods

### Overview

This study is reported in line with the CROSS checklist (Consensus-Based Checklist for Reporting of Survey Studies) [12] (Table S1 in Multimedia Appendix 1). We used a mixed methods survey design to obtain information from survey questions, both quantitative and semiqualitative, providing complementary information, within the context of a clinical feasibility trial in an NHS setting [13].

### Study Design and Participant Recruitment

Participants in the PD-Care program feasibility study were offered to participate in this wearable substudy. The primary aim of the PD-Care program is to develop and trial a supported self-management tool for people with PD, the full methods of which have been described previously [13]. The study emphasizes inclusive participation with broad inclusion criteria, including no age limit, inclusion of participants with cognitive impairment as long as they have the capacity to consent, and in any stage of PD. Remote delivery via video or telephone calls increased accessibility to study participation. Following the development of the intervention, a planned feasibility study was conducted before the randomized controlled trial (ISRCTN92831552). The feasibility study included 35 participants with assessments at baseline and post-intervention at 3 months. Participants were recruited through general practice mailouts, secondary care neurology teams, and the neurology research register.

After the 3-month follow-up assessments, all participants were offered to participate in a substudy, wearing 1 of 2 devices for a one-off 7-day period. After wearing and returning the device, participants were asked to provide feedback on the acceptability of the device via a telephone survey. The researcher entered the participant’s responses directly into the database while on the phone call. In addition, when downloading the data from the devices, the researcher recorded any errors that occurred. This substudy received input from the PD-Care patient and public engagement group, including where the device should be worn, how long it should be worn for, and which acceptability questions should be asked in the survey.

### Devices

Two different devices, ActivInsights [14] and Axivity AX3 [15] were used. The choice of the 2 devices was guided by pragmatic reasons for the purpose of a larger, publicly funded trial in the NHS. Both devices have been used in previous clinical research in patients with PD [14,15]. Both devices were worn at the wrist. In addition, as recommended by the patient and public advisory group, a sensor worn on the trunk was also tested to capture different clinical features. We therefore used 2 Axivity AX3 devices worn simultaneously, one on the wrist and the other on the trunk, whereas the ActivInsights device was worn on the wrist only.

The ActivInsights device provided output data on sleep, sedentary time, active time, and time spent exercising. The Axivity AX3 device output provided raw data on movement, recorded as a continuous wave accelerometer format, which was converted to a CSV file for analysis.

### Data Collection

The wearable devices and instructions were sent to participants in the post, with a request to return in the post after 7 days using a stamped addressed envelope. Seven days was chosen due to the limit on the battery life of the Axivity AX3 device while still collecting enough data to have an average over several days. Participants were allocated to a device depending on what was available once they had completed study follow-up assessments. We had 8 wearable devices of each ActivInsights and Axivity AX3, but as 2 devices were needed for each participant wearing Axivity AX3, fewer participants used this device. The date to start and stop recording for participants was preprogrammed into the device before posting out, and participants were asked to start and stop wearing the device on a specific date and time. Both devices recorded continuously, and we asked participants to inform us of the 7-day time period during which they wore the device, depending on when it arrived. No feedback from the devices was provided to participants. Data upload was undertaken by the researchers upon the return of the devices.

### Outcome Measures

#### Acceptability

The overall rate of participation in the substudy was recorded. After wearing and returning the device, participants were contacted via telephone to provide feedback about the acceptability of using their wearable device through a survey. We captured quantitative data to enable inferential statistical tests to be conducted to compare between devices as well as open-ended questions to ensure that the nuance of people’s individual experiences was understood.

The open-ended questions were (1) How did you find wearing the device? (2) How did you find putting the device on? (3) Did you take it off and why? (4) What was your overall impression? (5) Did you prefer the wrist- or trunk-worn device (Axivity AX3 only) and why? The quantitative assessments included questions using a Likert scale from 1 to 5 (1=strongly agree and 5=strongly disagree) whether (1) the device was comfortable to wear, (2) the device was easy to put on, (3) the device was easy to wear, (4) the device was embarrassing to wear, and (5) if they were happy to wear the device for longer than 7 days.

The survey was designed by the research team to cover the core components of wearable acceptance while being short and quick to administer. The survey was not based on any specific framework or guideline. A pragmatic approach with short survey questions was adopted to minimize participant burden, to reduce the risk of attrition in the context of the overall feasibility study, which involved collecting a wide range of outcome measures.

#### Technical Reliability

We assessed whether issues of battery shortage, recording failure, or upload difficulties impacted on the availability of usable data.

### Data Analysis

To ensure that the participants in the sample did not differ from those who chose to wear an activity monitor, the Fisher exact test and 2-tailed *t* test comparisons were conducted on demographic characteristics. To explore differences in acceptability between the different monitors, Mann-Whitney *U* tests and Wilcoxon matched pairs signed rank tests were conducted. All analyses were conducted in Stata (version 17; Stata Corp 2021). Open-ended question answers were written out, as participants provided their answers verbally, and responses were synthesized through content analysis to identify common concepts and views between participants.

### Ethical Considerations

Participants were taking part in the feasibility study of the PD-Care trial (ISRCTN92831552) and provided optional consent to take part in this wearable substudy. This substudy received a favorable opinion by the London Queen Square Research Ethics Committee and Health Research Authority approval (18/LO/1470) on October 31, 2018, as part of the feasibility study application. All participant’s data were pseudoanonymized through unique study ID numbers, and all study documentation, including wearable data and survey responses, were stored against this ID. Participants received a US $26 voucher for completing baseline assessments and US $13 voucher for completing 3-month follow-up assessments, as part of the feasibility study. No additional compensation was provided for taking part in this wearable substudy.

## Results

### Participants

**Figure 1.**
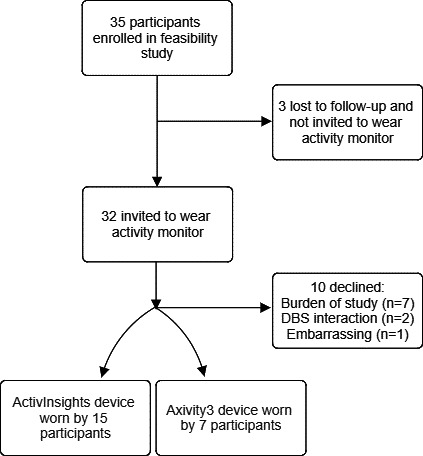
Flowchart of participants in the study. DBS: deep brain stimulation.

Between October 2020 and February 2021, 35 participants with a clinical diagnosis of PD and living in a community setting were recruited to the PD-Care program from several secondary care sites in the United Kingdom. Of these, 1 moved permanently to a care home and was no longer eligible, and 2 were lost to follow-up in the overall study. The remaining 32 were offered participation in the device substudy. The average age of the 32 participants invited to wear a wearable device was 68.2 (SD 11.3) years, 11 (34%) were female, and 23 (72%) were White British. Of these, 22 (69%) agreed to wear a device for 7 days. Reasons for not participating were participants feeling burdened from the feasibility study and did not want to provide more data (n=7), participants had a deep brain stimulation device fitted and were uncertain about potential interactions with the wearable (n=2), and finding it embarrassing to wear (n=1) (Figure 1).

There were no statistically significant differences in age, sex, or disease duration or severity between participants and nonparticipants (Table S2 in Multimedia Appendix 1). In total, 15 of the 22 participants were allocated the ActivInsights device (wrist only) and 7 were asked to wear the Axivity AX3 device (wrist and trunk). There were no statistically significant differences in the characteristics of those who wore the Axivity AX3 and those who wore the ActivInsights devices (Table S3 in Multimedia Appendix 1).

### Patient Acceptability

#### ActivInsights

Feedback to open-ended questions suggested that the device was easy to use, and participants quickly forgot they were wearing it (n=11). Some people commented that the strap was difficult to put on and needed help (n=5), and others suggested that it would have been nice if the device had a screen that gave them some feedback about their activity levels (n=3). Quantitative feedback suggested that overall the device was comfortable, easy to wear, and not embarrassing, and participants would be happy to wear it for longer than 7 days (Tables1  2 and Multimedia Appendix 2 ).

**Table 1.** People with Parkinson disease who responded in a positive way to the survey questions about their experience of wearing different wearable devices in the home for 1 week.

Device	It was comfortable, n (%)	It was easy to put on, n (%)	It was easy to wear, n (%)	It was embarrassing to wear^a^, n (%)	I would wear it for more than 7 days, n (%)
Axivity AX3 wrist (n=7)	5 (71)	5 (71)	6 (86)	7 (100)	4 (57)
Axivity AX3 trunk (n=7)	4 (57)	2 (29)	3 (43)	7 (100)	1 (14)
ActivInsights wrist (n=15)	15 (100)	10 (67)	15 (100)	15 (100)	13 (87)

a Percentage inversed.

**Table 2.** Mann-Whitney *U* tests comparing the acceptability responses between ActivInsights and both Axivity AX3 wrist and trunk and Wilcoxon matched pairs signed rank test comparing responses between Axivity AX3 wrist and trunk.

Device comparison	It was comfortable, *P* value	It was easy to put on, *P* value	It was easy to wear, *P* value	It was embarrassing to wear, *P* value	I would wear it for more than 7 days, *P* value
ActivInsights versus Axivity AX3 wrist	.02	.72	.048	N/A^a^	.02
ActivInsights versus Axivity AX3 trunk	.004	.38	.004	N/A	<.001
Axivity AX3 Wrist versus Axivity AX3 trunk	.50	.50	.25	N/A	.25

a N/A: not applicable.

#### Axivity AX3

All participants reported that they preferred the wrist-worn device to the trunk-worn device. Problems with the trunk-worn device centered around the tape not holding the device in place and having to be reapplied (n=7). This led to 3 of 7 participants not wearing the trunk-worn device but still provided feedback on both devices. The wrist-worn device was acceptable to all, with only a few comments about it being bulky and uncomfortable (n=3) and 1 participant wanted feedback on what the device was recording.

Quantitative feedback suggested that, on average, participants were neutral to the trunk-worn device being comfortable, easy to put on, and easy to wear but disagreed with being happy to wear it for longer than 7 days. For the wrist-worn wearable device, participants on average agreed that it was easy to put on and wear and were neutral about its comfort and whether they would wear it for more than 7 days (see Table 1 and Multimedia Appendix 2).

### Patient Acceptability—Comparison Between Groups

Using Mann-Whitney *U* tests to compare the acceptability of the devices, overall, the ActivInsights device was more acceptable than both the Axivity AX3 wrist- and trunk-worn devices. The ActivInsights device was rated as more comfortable than the wrist-worn and trunk-worn Axivity AX3 devices (n=15, 100% vs n=5, 71%; *P*=.02 and n=4, 57%; *P*=.004, respectively), rated as easier to wear (n=15, 100% vs n=6, 86%; *P*=.048 and n=3, 43%; *P*=.004, respectively), and rated as more suitable to wear more than 7 days (n=13, 87% vs n=4, 57%; *P*=0.02 and n=1, 14%; *P*<.001, respectively). Using Wilcoxon matched pair signed rank tests, there were no significant differences between the Axivity AX3 trunk-worn and wrist-worn device, but the numbers were small. There were no significant differences between all 3 devices for the ease to put them on (Table 2) and for embarrassing to wear, and all participants responded positively, stating the devices were not embarrassing.

### Technical Reliability

#### ActivInsights

There were no issues reported regarding battery shortage or recording failure. There were data upload errors with 3 (20%) of the devices, whereby the system failed when trying to upload the data, and when corrected, the data had been wiped from the device. Usable data were therefore available in 12 of 15 (80%) participants.

#### Axivity AX3

Of the 11 devices worn by 7 participants (7 wrist and 4 trunk), 6 (3 participants) were worn for shorter durations, resulting in missing data, for a median of 27 (IQR 8‐48) hours, and this was due to hospitalization (n=1) and participant forgetting (n=2). In 3 other devices, the device ran out of battery during the data collection period. Despite the errors, some usable data was available for all participants with this device.

## Discussion

### Principal Findings

We assessed the acceptability and technical feasibility of 2 wearable devices used in trials with people with PD, which showed that wearable devices are acceptable to people with PD and feasible to administer and collect data with. Some data errors were found due to battery life, participant utility, and upload issues. Although several studies using wearable devices have reported validity for recording human movement in a free-living environment [16], including in people with PD [2,17,18], successful clinical adoption in monitoring movement in PD depends on acceptability and technical reliability [5,17]. To date, there is no fully validated system to monitor clinical features or activities in PD [5]. The bulk of data on technical reliability with the use of individual wearable devices comes from the studies led or supported by manufacturers of these devices [16]. This is the biggest study to date that reports the feedback of patients with PD from wearing 2 different monitoring devices using open and closed questions.

### Patient Acceptability

Feedback from patients suggests that monitoring using both ActivInsights and Axivity AX3 is mostly acceptable, although there is a proportion of patients who do not wish to use a monitoring device due to burden and embarrassment. The burden was due to participants having to complete clinical assessments as part of the feasibility study. If the participants had had the option of clinical assessments or activity monitors, then uptake may have been higher. There were no demographic characteristics that differed between the participants who chose to be monitored and those who chose not to be monitored, including sex, age, and deprivation level. Largely, our findings are in line with those from a US population with PD, using a different device, suggesting ease of use and willingness to wear the device over a longer period when located on the wrist [9]. In addition, these findings reflect acceptability in other UK patient groups, such as wrist-worn alcohol sensors in those who are alcohol dependent, where people were positive about the devices, despite the perceptions of health care professionals that service users may have challenges using them [19].

We did not specifically examine motivational factors, but another study has reported on the motivations for and barriers against monitoring PD symptoms among patients with PD [20]. Key motivations include the wish to discuss findings with health care providers, obtain insight into the effect of medication and other treatments, and follow the progression of the disease. Key barriers were not wanting to focus too much on having PD, symptoms being relatively stable, and lacking an easy-to-use tool [17]. As all participants in our study were monitored as part of a research intervention, these motivating factors mentioned may have been less directly relevant. As no clinical feedback or reports were provided to participants, and the purpose was to supplement clinical assessments in a trial, the setting and motivation will differ from those who would consider monitoring for a clinical purpose. Nevertheless, 4 (18%) of the participants expressed the wish to know what was being monitored.

Social embarrassment and the feeling that wearing a device for monitoring signaled a person as “old” have been highlighted as major factors affecting the acceptability of body-worn sensors [11]. Similarly, the use of a wearable device was embarrassing in some of our study participants, resulting in them declining participation in this substudy. In another study using Axivity AX3 on each wrist, the participants wore the device for 4 hours in a research facility and then for 1 week at home. Overall, the study reported that monitoring with body-worn sensors was acceptable to patients with PD and that most participants were willing to wear the sensor both at home and in public [11]. Concerns regarding safety particularly with deep brain stimulation could also be an additional barrier that could be resolved with better communication and technical support.

In addition, overall, the wrist-worn devices were more acceptable than the trunk-worn devices, as many participants found the trunk-worn device to be uncomfortable and difficult to keep on. However, there may also be issues with wrist-worn devices. A previous study using Axivity AX3 on each wrist reported that, for prolonged wearing, participants were less likely to agree that the sensors were comfortable to wear [11]. Responses to open-ended questions revealed that the main source of sensor discomfort related to the strap [11]. Though the acceptance of the strap on the 7-day monitoring was not a problem in our cohort, 23% (n=5) needed help with the strap to put the device on. Ideally, if the wearable device is to be worn by a patient with PD, they should be able to do this independently despite poor dexterity.

### Technical Reliability

The charging and preparation of the devices takes time and effort, with human errors leading to incomplete charging of the devices, and some participants forgetting to turn on the devices. The battery life on a single charge may not be long enough for long-term monitoring. There were also upload errors, with the upload failing in one-fifth of the participants using the ActivInsights device. Almost all wearable devices rely on a local computer interface, a stable internet connection for upload, and often an external storage system before being analyzed [2]. The upload to a cloud server relies on a good internet connection and can be a limiting factor in some settings [4]. Such technical problems not only add to costs and time pressure but can also lead to further anxiety for participants who might already have nonmotor symptoms of anxiety. More data are needed on failure rates with individual devices. On most occasions, device failure was identified after the recording had been completed, and the wearable devices had been sent back. Ideally, troubleshooting tools or a live screen display, as 4 of our participants suggested, could be more acceptable and help avoid recording failures. Furthermore, additional training of research staff and patients to ensure data upload could be helpful to overcome this issue.

### Study Limitations and Future Research

The duration of monitoring used in this study was short, and we had a small sample size. Studies with larger sample sizes and longer monitoring periods are needed. As part of the PD-Care program of research, a large randomized controlled trial is now underway including a substudy using wearable devices in people with PD. These data will be used to further validate the use of wearable devices as a clinical measure of PD symptoms as well as confirm the acceptability findings found in this feasibility study. Researchers should also explore differences in acceptability in people from underserved groups to ensure that the implementation of these devices does not widen health inequalities.

### Conclusions

The study highlights some issues that can be addressed by device manufacturers such as difficulty with strapping single-handedly in someone with limited dexterity, help in uploading data, and clearer instructions. We found better acceptability of the ActivInsights device by our participant cohort, but there was a higher rate of usable recording when using Axivity AX3. The prolonged use of devices worn on the trunk was less acceptable. More real-world experience, including patient feedback and multidisciplinary coordinated effort involving all key stakeholders, is critical before the widespread adoption of wearable devices for monitoring PD in clinical trials.

## Supplementary material

10.2196/63704 Multimedia Appendix 1 The CROSS (Consensus-Based Checklist for Reporting of Survey Studies) checklist and demographic characteristic comparisons.

10.2196/63704 Multimedia Appendix 2 Graph showing responses to each question, for each device.

## Abbreviations

CROSS: Consensus-Based Checklist for Reporting of Survey Studies
NHS: National Health Service
PD: Parkinson disease
