# Supplementary material for: Patient Acceptability and Technical Reliability of Wearable Devices Used for Monitoring People With Parkinson Disease: Survey Study
Source: JMIR Form Res. 2025 Mar 25;9:e63704. doi: 10.2196/63704 (PMC11962569; doi:10.2196/63704)
Supplement: Supplementary file 2 [file formative-v9-e63704-s002.docx]

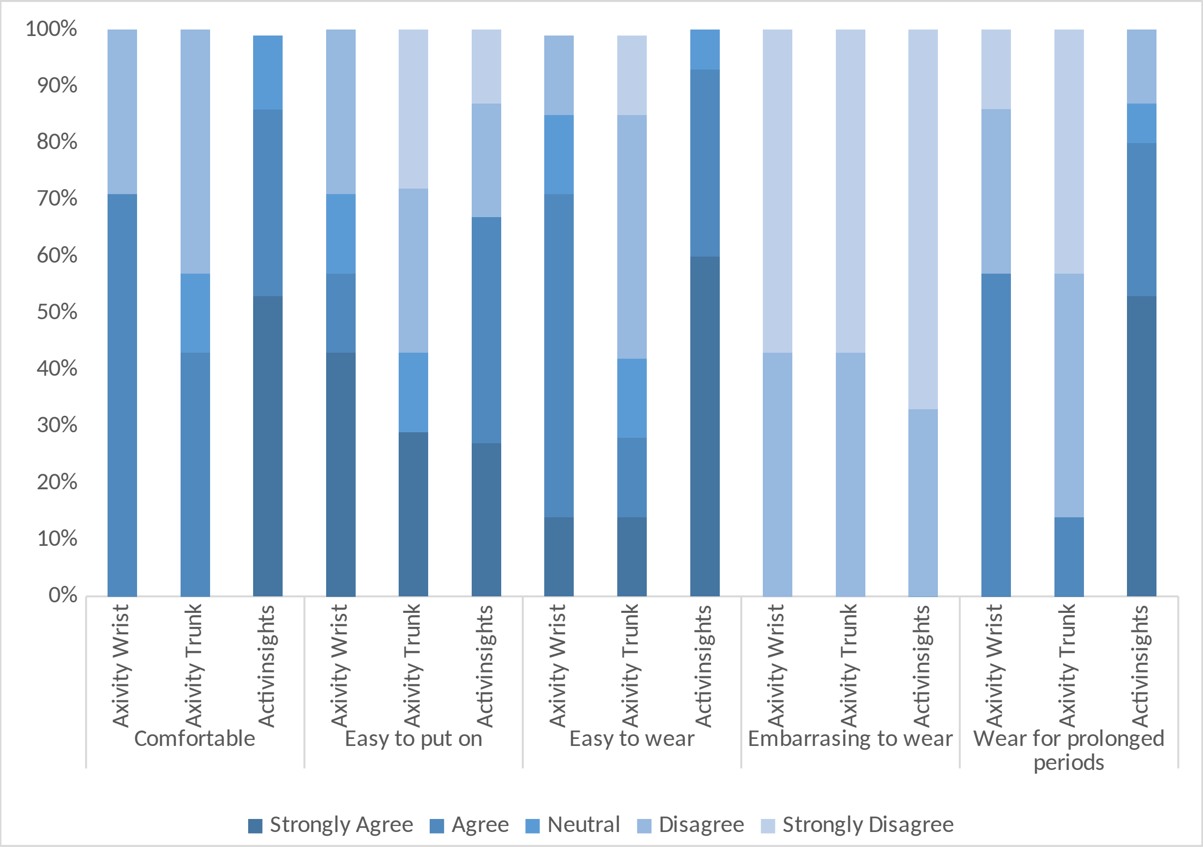

100%
90%
80%
70%
60%
50%
40%
30%
20%
10%
0%
Axivity Wrist
Axivity Trunk
Activinsights
Comfortable
Easy to put on
Easy to wear
Embarrasing to wear
Wear for prolonged periods
Strongly Agree
Agree
Neutral
Disagree
Strongly Disagree
